# Highlight: Changing the Canon—Revisiting a Classic Experiment to Alter the Genetic Code

**DOI:** 10.1093/gbe/evu053

**Published:** 2014-03-26

**Authors:** Danielle Venton

It doesn't take much for an organism to accept something radically new into its life. That is the conclusion of a recent inquiry into the mutability of life's fundamental genetic code by Ting-Fung Chan, a computational biologist at the Chinese University of Hong Kong and his colleagues.

“There are not actually that many genes involved in the change,” says Chan. For an organism to switch from using one amino acid to another, thereby permanently altering its genetic code, takes on the order of 20 genes. That, he says, is less than many in his field expected and “was one of the many surprises we had along the way.”

Chan and his colleagues, at the Chinese University of Hong Kong and the Hong Kong University of Science and Technology, recently completed an examination of the near-30-year-old “codon coevolution hypothesis” initially proposed by Jeffrey Tze-Fei Wong. Wong is among the coauthors of the new study in *Genome Biology and Evolution* (Wong 1975).

Wong proposed that the genetic code is an “imprint of the biosynthetic relationships between amino acids,” meaning that the organization of the genetic code is a reflection of an evolutionary process, as amino acids were incorporated gradually into proteins used for life’s functions.

Though many love to revel in the diversity of life, judged at another scale, life is surprisingly similar the world over. All cellular life encodes its genetic material with double-stranded nucleic acid chains, composed of the same four bases. A trio of these bases code for a particular amino acid, the same code being used the world over. A scant 20 amino acids, out of the hundreds of those possible, are used in the construction of proteins. These few amino acids are all that are used to make proteins, whether looking at a jellyfish, a redwood tree, or a fungal pathogen.

How did it get like this? Can organisms be induced to incorporate new amino acids? These are fascinating questions, says Chan. “If this is the result of evolution,” he says, “then perhaps it could be changed in the future.”

Wong went on to induce evolutionary changes in bacterial strains. He slowly weaned *Bacillus subtilis*, a common soil bacterium, on to 4-fluorotryptophan (4FTrp) and off canonical tryptophan in a strain called HR23.

In their recent work, Yu et al. (2014) wanted to know how this genetic leap had been possible. However, simply comparing the genomes of HR23 and wild-type *B. subtilis* would have presented a confusing picture.

“You'll see random genetic changes that may have nothing to do with the [new] phenotype,” says Chan. “The beauty of the study is that Jeffrey [Wong] did keep all these intermediate strains and allowed us to sequence them so that we would know what happened as a gradual process.” The researchers also sequenced and compared a HR23 precursor strain able to use both Trp and 4FTrp as well as a later strain of HR23 that regained the ability to use Trp.

They found a suite of differences, ranging from genes related to Trp metabolism and stress response to mutations in the transcription and translation machinery.

“Wong's original paper is a landmark in my mind,” says Jamie Bacher, a CEO of a biotech company in the San Francisco Bay Area. “It opened up a lot of questions and opportunity. Seeing the follow-up, seeing those original strains sequenced and compared is creative and exciting.”

He believes that the team’s findings may be practically relevant as well as theoretically interesting.

“I think there is the potential for this to be industrially applied,” Bacher says. “For example, they noted that one of the first mutations in the precursor strain was in the *mtrB* gene, a negative regulator of Trp transport.” 

Likely, *mtrB* opens up the transport of tryptophan in all forms, he says. “So if you’re going to optimize an industrial strain of bacteria to incorporate an unnatural amino acid, this kind of knockout mutation might be something you want to do early on.”

For their part, Chan and colleagues (Yu et al. 2014) currently focused on experimental validation, testing which of the observed mutations are the most important for conferring the ability to incorporate 4FTrp instead of Trp using a genome-editing approach.

Jeffrey Wong, Chan says, is naturally following the progress with interest. Thirty years ago, Wong guessed that, if the genetic codon table is the result of changes, then he could change it further. “He thought the changes would involve only a limited number of genes. After 30 years, he has a list in his hands.” Wong's response? “He's elated.”
